# Isolated posterior fossa involvement of progressive multifocal leucoencephalopathy in HIV: A case series with review of the literature

**DOI:** 10.4102/sajr.v21i2.1262

**Published:** 2017-11-14

**Authors:** Adziambei Mudau, Farhana E. Suleman, Clara M. Schutte, Zarina I. Lockhat

**Affiliations:** 1 Department of Radiology, University of Pretoria, South Africa; 2 Department of Neurology, University of Pretoria, South Africa

## Abstract

Progressive multifocal leucoencephalopathy (PML) is a progressive demyelinating condition resulting from infection with the John Cunningham virus and precipitated by immunocompromised states. The HIV pandemic, especially in sub-Saharan Africa, has resulted in an increase in the number of patients presenting with PML. Imaging plays an important role in diagnosis and the distribution of the disease is predominantly supratentorial. Isolated posterior fossa involvement is a rare finding with very few cases described in the literature. We present the largest case series of patients described in the literature, with isolated posterior fossa involvement of PML, in HIV-positive patients.

## Introduction

Progressive multifocal leucoencephalopathy (PML) is a demyelinating disorder, which results from opportunistic infection of the central nervous system (CNS) with the John Cunningham (JC) virus. This virus is believed to infect up to 80% of the human population prior to adulthood without producing obvious illness, and it remains latent until reactivation by an immunodeficient state.^1^ HIV infection is the most common predisposing factor for symptomatic JC virus disease, and the HIV pandemic has resulted in an increased prevalence of PML, affecting 3% – 7% of HIV-infected individuals before the highly active antiretroviral therapy (HAART) era.^2^ Although the frequency of PML has decreased with HAART, a more significant decrease has been noted with other CNS opportunistic infections.^3^ PML is recognised as an AIDS-defining illness, and the vast majority of HIV-infected patients with PML have CD4 lymphocyte counts < 200 cells/mm^3^. JC virus reactivation can, however, also occur in other patients with compromised immunity as well as in patients on chronic immunosuppressive therapy.

There are several studies that have demonstrated a high sensitivity and specificity of cerebrospinal fluid (CSF) polymerase chain reaction (PCR) for JC virus in PML.^3^ Many authorities regard the demonstration of JC viral DNA coupled with the appropriate clinical and radiologic features sufficient to be diagnostic of PML.^4^ Table 1 summarises the diagnostic criteria for PML.^4^ The diagnosis can also be confirmed with tissue histopathology after brain biopsy.

**TABLE 1:** Diagnostic criteria for progressive multifocal leucoencephalopathy^4^: Clinical, radiographic and laboratory data.

Certainty of diagnosis	Compatible clinical features	Compatible imaging features	CSF PCR for JC virus
Definite	+	+	+
Probable	+	−	+
	−	+	+
Possible	+	+	-/not done
	−	−	+
Not PML	−	−	−
	+	−	−

PML, progressive multifocal leucoencephalopathy; CSF, cerebrospinal fluid; PCR, polymerase chain reaction; JC virus, John Cunningham virus.

## Imaging features

Typically PML is a confluent, bilateral but asymmetric, supratentorial white matter disease.

Although lesions may develop in any area of the brain, they are most common in the subcortical white matter and U-fibres, favouring the frontal and parietooccipital regions. PML can be unilateral or rarely there may be only a single lesion.^2^ The lesions exhibit no mass effect and infrequent contrast enhancement.^3^ There is no involvement of the optic nerves or spinal cord. Isolated posterior fossa involvement is rare, but has been described.^5,6^

Clinical presentation is non-specific with focal neurologic deficits dependant on the lesion location. Symptoms worsen over time as the lesions progress. Most often, PML presents with motor deficits, visual disturbances and cognitive impairment, in a subacute evolution.^7^

With regard to imaging, computed tomography (CT) scan of the brain in patients with PML may show hypodense lesions. These lesions exhibit no mass effect and infrequently enhance post contrast.^1,3^ Magnetic resonance imaging (MRI) is, however, more sensitive to the white matter lesions of PML. On MRI, PML demonstrates hyperintense lesions on T2-weighted and Fluid attenuation inversion recovery (FLAIR) sequences; hypointense signal on T1-weighted sequences and no mass effect, despite their size.^1,3^

Enhancement can be seen with PML associated with immune reconstitution inflammatory syndrome (PML-IRIS), which occurs as the immune system recovers in patients commenced on HAART. It is reported to account for up to 18% of PML in HIV-infected patients.^8^ Tan et al. noted contrast enhancement in 56.7% of PML-IRIS cases.^9^ Enhancement suggests an inflammatory component, but absence of enhancement does not exclude the diagnosis.

Diffusion-weighted imaging (DWI) has been related with the stage of the disease. Active lesions demonstrate an incomplete rim of restricted diffusion, with high apparent diffusion co-efficient (ADC) values in the centre of the lesion. No restricted diffusion is appreciated with older lesions. A larger central core is associated with a worse prognosis.^10^

## Case series

### Case 1

**FIGURE 1:**
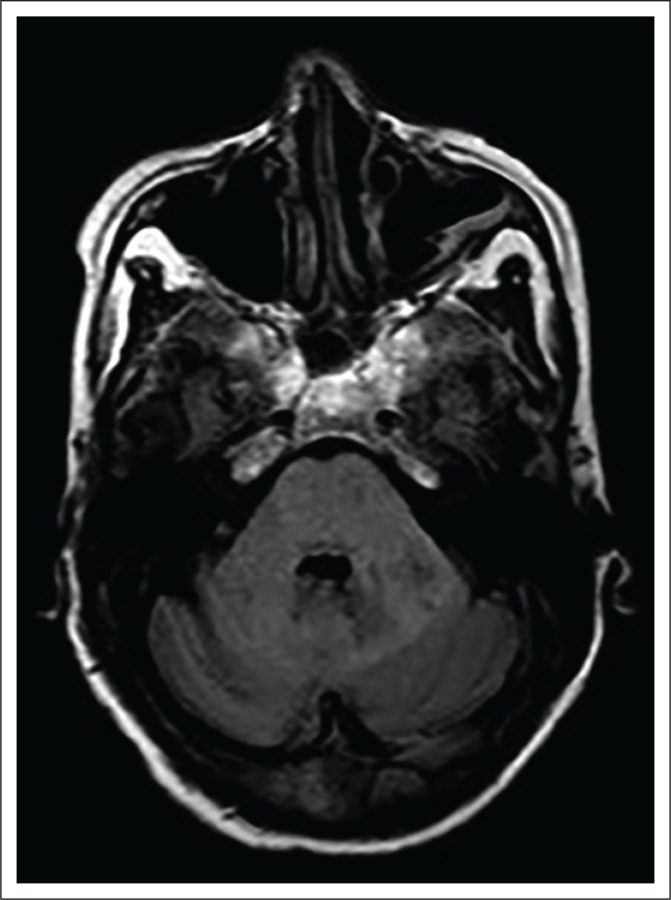
Axial FLAIR image of the patient discussed in Case 1, at the level of the pons demonstrating high-signal in the entire pons and part of the left cerebellar hemisphere. Images at a lower level (not shown) demonstrated more extensive involvement of the left cerebellum.

A 34-year-old HIV-positive woman with a CD4 cell count of 24 cells/µL and not on HAART, presented with confusion and ataxia. On examination, she had nystagmus, severe dysarthria and a broad-based gait. She had a history of alcohol abuse, and the possibility of Wernicke encephalopathy was considered, which was treated with thiamine. MRI was requested, which showed high-signal on T2-weighted and FLAIR sequences in the left cerebellar hemisphere, pons and midbrain (Figure 1). There was no restricted diffusion, no associated mass effect nor enhancement on post contrast imaging. The CSF yielded no growth, and the JC virus PCR was positive. The patient deteriorated in the ward and subsequently demised.

### Case 2

**FIGURE 2:**
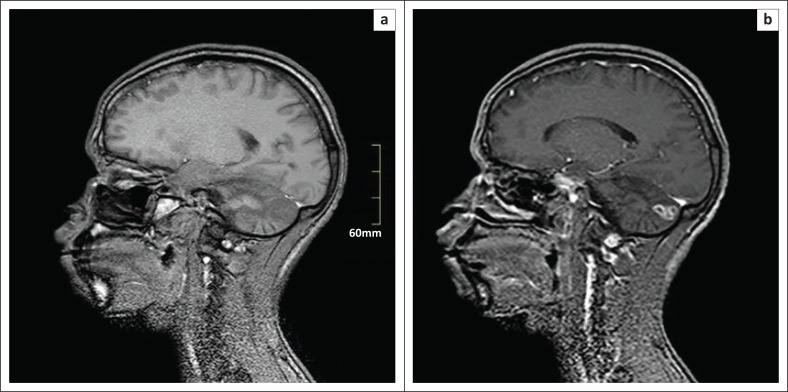
Sagittal pre-(a) and post-contrast (b) T1W images of the patient discussed in Case 2 at the level of the right cerebellar hemisphere demonstrating low signal intensity in the cerebellum with enhancement in the same region post-contrast administration.

A 34-year-old HIV-positive man on HAART presented with 1-month history of cerebellar ataxia. His CD4 count was 30 cells/µL. A CT scan of the brain showed hypodensity in the left cerebellar hemisphere and generalised cerebral atrophy. MRI was requested, which showed multiple high-signal changes on T2-weighted imaging involving the pons and right cerebellar hemisphere with some enhancement post contrast (Figure 2). No restriction was noted on DWI. Tests for toxoplasmosis were negative, and CSF yielded no growth. The JC virus was positive in the CSF. The patient was treated with triple antibiotic therapy, as well as anti-tuberculosis medication. The possibility of a CNS IRIS was considered, and a course of steroids was added. The HAART was optimised and the patient was discharged to a rehabilitation unit.

### Case 3

**FIGURE 3:**
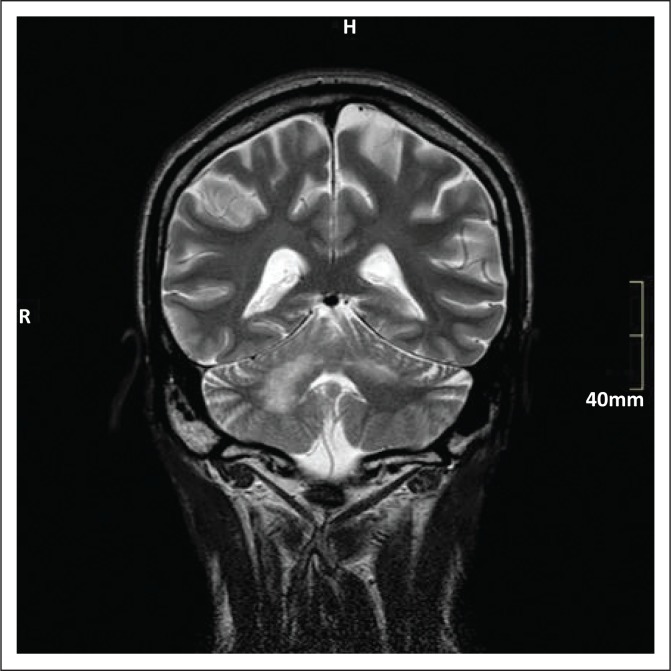
Coronal T2 weighted image of the patient discussed in Case 3, showing high-signal in both cerebellar hemispheres with greater involvement of the right hemisphere.

A 40-year-old HIV-positive male patient on HAART and anti-tuberculosis medication presented with 3-week history of loss of balance, dizziness and difficulty in speaking. On examination, he had ataxia, nystagmus, dysmetria and dysdiadocokinesis. His CD4 count was 167 cells/µL. MRI showed high-signal on T2-weighted and FLAIR images bilaterally in the cerebellar hemispheres with the right more involved compared with the left (Figure 3). The lesions did not demonstrate any restricted diffusion or enhancement post contrast. The CSF was not active and demonstrated no growth. The patient’s anti-tuberculosis medication was continued, and the HAART was optimised. He was discharged to the rehabilitation unit.

### Case 4

**FIGURE 4:**
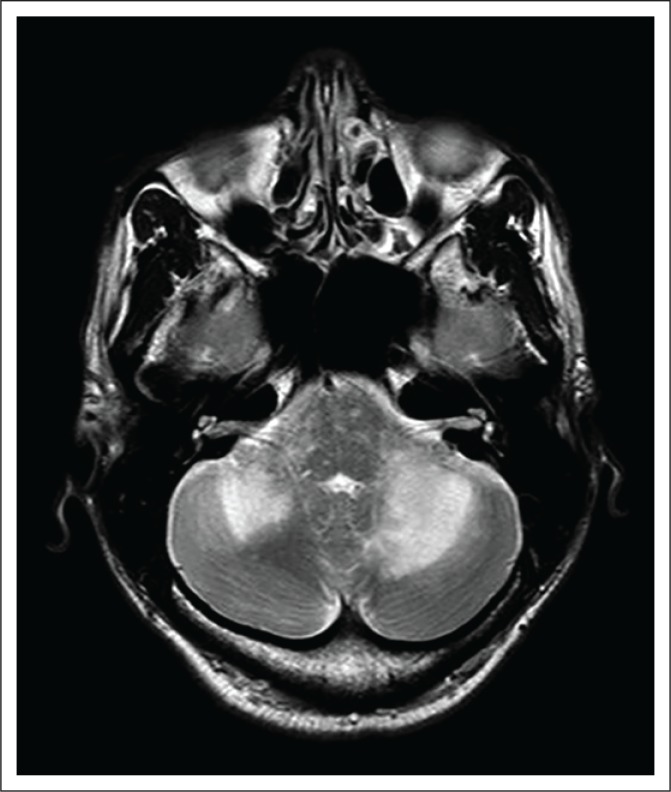
Axial T2 weighted image of the patient discussed in Case 4, at the level of the cerebellar pontine angles demonstrating confluent high-signal changes in both cerebellar hemispheres and patchy changes in the pons.

A 38-year-old male patient, HIV-positive with a CD4 count of 143 cells/µL, presented with left hemiparesis, diplopia and ataxia. He was not on HAART. A CT scan of the brain showed asymmetric hypodensities in the cerebellum with no enhancement post contrast. MRI showed bilateral confluent non-enhancing, cerebellar high-signal changes on T2-weighted and FLAIR sequences with patchy changes in the brainstem (Figure 4). The patient was advised regarding antiretroviral therapy (ART) and was discharged.

### Case 5

**FIGURE 5:**
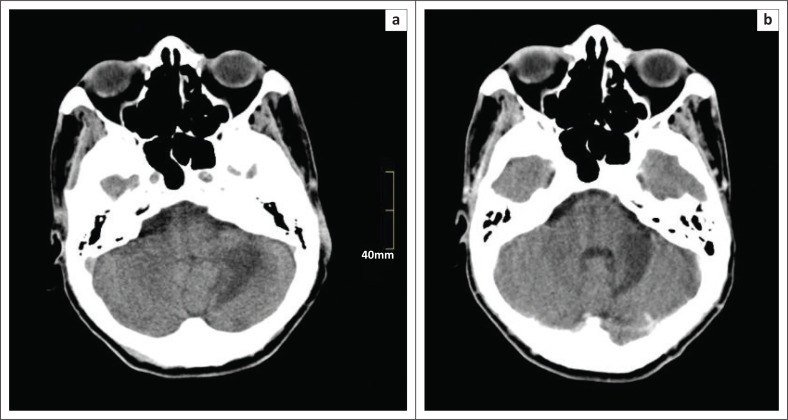
Axial (a) pre- and (b) post-contrast CT scan of the patient discussed in Case 5, demonstrating low density in the left cerebellar hemisphere with no contrast enhancement of the lesion.

A 35-year-old male patient presented with dizziness, speech problems and cerebellar ataxia. He was HIV-positive with a CD4 count of 90 cells/µL. He was not on HAART and anti-tuberculosis treatment for pulmonary tuberculosis. On examination, he was chronically ill with nystagmus, dysarthria, dysmetria, ataxia and left-sided hemisensory loss. JC virus was found on examination of the CSF. CT scan demonstrated low density in the left cerebellar hemisphere, which did not enhance on the post contrast imaging (Figure 5). T2-weighted and FLAIR sequences on MRI demonstrated high-signal changes in the left cerebellar hemisphere, left superior cerebellar peduncle and diffusely in the pons. No enhancement or restricted diffusion was noted. The patient was discharged to the rehabilitation unit.

### Case 6

**FIGURE 6:**
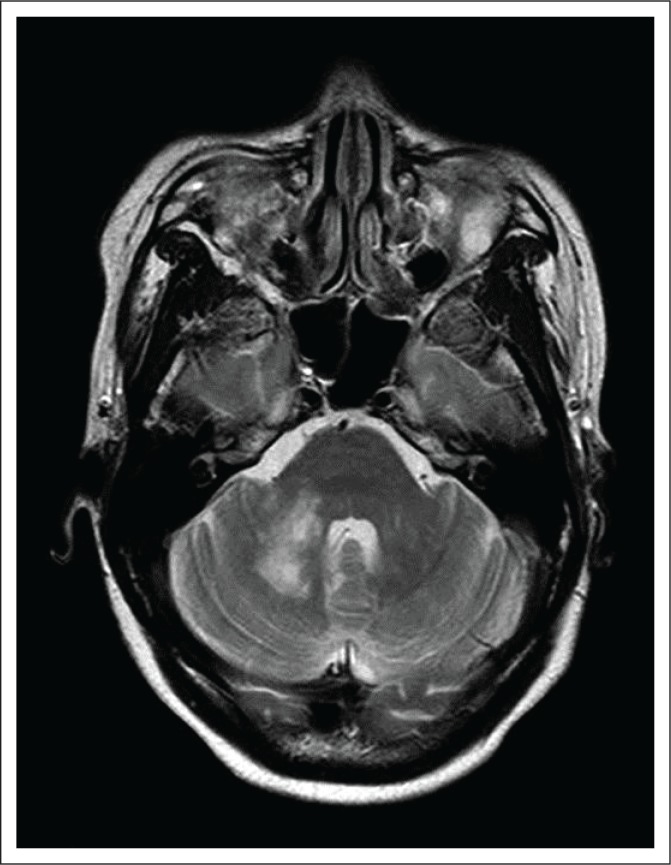
Axial T2 weighted image of the patient in Case 6, showing high-signal changes in the right cerebellum and cerebellar peduncle.

A 38-year-old HIV-positive woman, with a CD4 count of 28 cells/µL, on HAART, presented with progressive right-sided dysmetria and ataxia. The patient was pregnant at 30 weeks gestation. CT showed low density area in the right cerebellar hemisphere, which was shown on MRI to extend into the right cerebellar peduncle, pons and medulla (Figure 6). No contrast was administered because of the pregnancy. No restricted diffusion was noted. CSF studies revealed no abnormalities except positivity of JC virus. The patient underwent an emergency caesarean section for foetal distress and was discharged back to the referral hospital.

Table 2 summarises the clinical and MRI findings of the patients presented in the case series.

**TABLE 2:** Clinical and magnetic resonance imaging findings of the patients.

Case	Age (years)	HIV	CD4(10^6^/L)	JC PCR in CSF	Cerebellar involvement	Brainstem involvement
1.	34	Positive	24	Positive	Left cerebellar hemisphere	Midbrain and pons
2.	34	Positive	30	Positive	Bilateral	Pons
3.	40	Positive	167	Positive	Bilateral	-
4.	38	Positive	143	Positive	Bilateral	-
5.	35	Positive	90	Positive	Left cerebellar hemisphere	Pons
6.	38	Positive	28	Positive	Right cerebellar hemisphere	Pons and medulla

CSF, cerebrospinal fluid; PCR, polymerase chain reaction; JC, John Cunningham.

## Discussion

In 2015, the World Health Organization (WHO) reported that 36.7 million people were living with HIV.^11^ Sub-Saharan Africa remains the most severely affected, with nearly 1 in every 25 adults (4.4%) infected and accounting for nearly 70% of the people living with HIV worldwide.^11^ The estimated overall HIV prevalence rate is approximately 12.7% of the total South African population. The total number of people living with HIV in South Africa is estimated at approximately 7.03 million in 2016.^12^

The HIV pandemic led to a new population of immunocompromised patients. Studies suggest that HIV accounts for approximately 80% of the PML cases.^2^ The parietal lobe is the most commonly involved, followed by the frontal lobe. Supratentorial lesions typically involve the subcortical white matter with a scalloping appearance.^2^ White matter of the posterior fossa is the next common area of involvement; however, isolated cerebellar white matter or isolated medullary involvement is less common.^2^

One study of 47 HIV-positive patients with PML reported only two patients with isolated posterior fossa involvement.^1^ In this article, we describe a case series of six patients, from a South African tertiary hospital, with isolated posterior fossa involvement of PML in HIV-positive patients. All patients tested positive for JC PCR in the CSF.

Imaging plays a pivotal role in the diagnosis and follow-up of patients with PML.^2^ MRI is the technique of choice for evaluation of this condition. All our patients had cerebellar involvement and no supratentorial involvement. Post contrast enhancement was demonstrated in only one of the patients.

Progressive multifocal leucoencephalopathy can be differentiated from HIV encephalopathy, which shows a combination of atrophy and symmetric periventricular or diffuse white matter disease on imaging.^13,14^ PML usually spares the periventricular and deep white matter and causes no atrophy in the active phase of the disease.^2^ In cytomegalovirus encephalitis, the distribution of white matter lesions is periventricular, and subependymal enhancement is seen.^13,14^

More recently, PML has been associated with the administration of natalizumab, an immunomodulating monoclonal antibody, for the treatment of multiple sclerosis. Given the presence of two demyelinating disorders, the diagnosis of new lesions and PML are challenging. Similar to PML-IRIS with HAART, IRIS can also develop in multiple sclerosis, once natalizumab is discontinued.^15^

The vast majority of HIV-infected patients with PML have a CD4 lymphocyte count of < 200 cell/mm^3^.^4^ CSF examination is very useful in excluding other diagnosis, however, the greatest value of CSF analysis is demonstrating the presence of JC virus by PCR.^4^ JC virus assay is positive in approximately 70% – 90% of patients not taking ART but only as few as 60% of patients on ART.^13^ All our patients had a positive JC virus PCR.

The main treatment approach in HIV-related PML involves combination ART. The introduction of more potent antiretroviral regimens has led to a considerable decrease in incidence and mortality of PML.^13^

Progressive multifocal leucoencephalopathy in HIV-infected patients is associated with a poor prognosis, with approximately 50% mortality within 2 years from disease onset.^16^ This factor is totally dependent on co-morbid diseases in HIV-infected patients.

## Conclusion

We describe a series of six patients with PML with isolated posterior fossa involvement. In our literature search, there were only seven reported cases of PML isolated to the posterior fossa in HIV-positive patients.^4,10^ In one review of 47 patients with proven PML, only two had isolated posterior fossa involvement.^1^ The high number in our series is probably because of the high incidence of HIV infection in our region as well as the difficulties experienced by large numbers of patients in accessing adequate treatment.
